# Local Delivery and Controlled Release Drugs Systems: A New Approach for the Clinical Treatment of Periodontitis Therapy

**DOI:** 10.3390/pharmaceutics15041312

**Published:** 2023-04-21

**Authors:** Mariacristina Amato, Simona Santonocito, Alessandro Polizzi, Gianluca Martino Tartaglia, Vincenzo Ronsivalle, Gaia Viglianisi, Cristina Grippaudo, Gaetano Isola

**Affiliations:** 1 Department of General Surgery and Surgical-Medical Specialties, School of Dentistry, University of Catania, 95124 Catania, Italy; 2 UOC Maxillo-Facial Surgery and Dentistry, Department of Biomedical, Surgical and Dental Sciences, School of Dentistry, Fondazione IRCCS Ca’ Granda, Ospedale Maggiore Policlinico, University of Milan, 20100 Milan, Italy; 3 Department of Head and Neck, Division of Oral Surgery and Implantology, Catholic University of the Sacred Heart, Fondazione Policlinico Gemelli IRCCS, 00168 Rome, Italy

**Keywords:** local delivery drugs, pharmaceutics, natural drugs, antibiotics, controlled released drugs, inflammation, periodontitis, nutraceutical agents, drugs, agents

## Abstract

Periodontitis is an inflammatory disease of the gums characterized by the degeneration of periodontal ligaments, the formation of periodontal pockets, and the resorption of the alveolar bone, which results in the destruction of the teeth’s supporting structure. Periodontitis is caused by the growth of diverse microflora (particularly anaerobes) in the pockets, releasing toxins and enzymes and stimulating the immune system. Various approaches, both local and systemic, have been used to treat periodontitis effectively. Successful treatment depends on reducing bacterial biofilm, bleeding on probing (BOP), and reducing or eliminating pockets. Currently, the use of local drug delivery systems (LDDSs) as an adjunctive therapy to scaling and root planing (SRP) in periodontitis is a promising strategy, resulting in greater efficacy and fewer adverse effects by controlling drug release. Selecting an appropriate bioactive agent and route of administration is the cornerstone of a successful periodontitis treatment plan. In this context, this review focuses on applications of LDDSs with varying properties in treating periodontitis with or without systemic diseases to identify current challenges and future research directions.

## 1. Introduction

Periodontitis is a chronic inflammatory disease affecting tooth-supporting tissues, manifesting by probing depths (PD), loss of clinical attachment level (CAL), and alveolar bone resorption. It is a multifactorial disease caused by an alteration of the microbiota of the gingival pockets and an abnormal immune response of the host. It can get worse in the presence of other factors, such as systemic diseases, such as diabetes, or bad habits, such as smoking. It is spread worldwide, affecting 10–15% of the world’s population, according to WHO’s estimations. Due to its multifactorial etiology, patients must be treated with personalized therapy, having control of the infection as the main objective.

According to the guidelines by Sanz et al. [1], the current standard treatment of stages I, II, and III of periodontitis is based on the following steps: motivation and education of the patient to improve domiciliary oral hygiene; control of other factors that enhance the periodontal disease, including diabetes, cardiovascular disease, and/or smoking cessation; and subgingival instrumentation performed by the clinician. In addition to the above-mentioned therapeutical interventions, according to Herrera et al. [2], Stage IV of periodontitis requires surgical correction of the bone defects and multidisciplinary intervention, such as temporary control of secondary occlusal trauma, orthodontic therapy, rehabilitation of the edentulous spaces, and tooth-supported or implant-supported dental prostheses (Table 1).

Supragingival and subgingival instrumentation, also known as scaling and root planing (SRP), and rigorous home dental care are essential to control the infection. In fact, since the pathogenic bacteria are organized in biofilm, the above-mentioned procedures remove the etiological microbiological factor of periodontitis. In addition to the mechanical removal of the biofilm, adjunctive systemic or local host-modulating agents can help in the treatment of periodontal disease [1]. To obtain the best results associated with the lowest risk from the adopted pharmacological therapy, it is important to modulate the molecule release. For this reason, a local drug delivery system (LDDS) has been suggested to be adopted in association with SRP (Table 1). The use of LDDSs is a promising therapeutic strategy applied in a variety of medical fields, including the treatment of local infections of the vagina, nose, eye, and skin [38]. In the treatment of periodontitis, it has been demonstrated that it is very important to maintain an effective drug concentration in the periodontal pockets for a sufficient timespan [39]. For this reason, an LDDS is a precious tool for local adjunctive pharmacological periodontal therapy.

The aim of this review is to identify current challenges and future research directions focusing on applications of LDDSs with varying properties in the treatment of periodontitis with or without systemic diseases.

## 2. Materials and Methods

To include articles in this review, we used the following search engines: PubMed and Google Scholar. We used the following keywords: local drug delivery, LDDS, and periodontitis. The total amount of articles found were: 868 on PubMed and 142 on Google Scholar, which were published from 1979 to 2022. Among the founded articles, we included 131 in this review, after excluding papers that did not respect the criteria for selection of papers and duplicate papers. At least two independent researchers reviewed titles and abstracts for inclusion. After the first analysis, full texts of the articles were requested, which were evaluated by two researchers to assess final inclusion/exclusion. If there was disagreement between the two researchers, a third researcher’s opinion was requested, and the decision was taken by consensus. The first analysis was based on the following standards: RCTs, cohort studies, case–control studies, and case–series that included, at least, a sample number of 15, meta-analysis, and a systematic review. The language of publication was not a feature that limited the inclusion of studies.

## 3. Drug Delivery System

The drug delivery system is a central point in pharmacological therapies. During the past years, the scientific community has focused on the variety of molecules that can control drug release competing with the ordinary methods of administration. Many medical fields have gained advantages using controlled LDDSs, which have been investigated and applied in oncology, cardiology, and ophthalmology [40,41,42,43,44]. In fact, thanks to the development of controlled drug delivery systems, problems normally associated with conventional drug delivery (such as syrup, capsules, tablets, etc.) are avoided. These problems are frequently: low bioavailability due to some drugs’ rapid excretion and hepatic metabolism and the need for frequent doses for drugs with short biological half-lives [45]. By controlling drug release, instead, it can be possible to maintain a sufficient quantity of the drug for a sufficient time lapse; in this way, it will achieve the desired therapeutic response, reducing the frequency of dosing and the collateral effects associated with a bigger amount of drug assumed by conventional administration (Table 2).

## 4. LDDS and Periodontal Treatment

**Figure 1:**
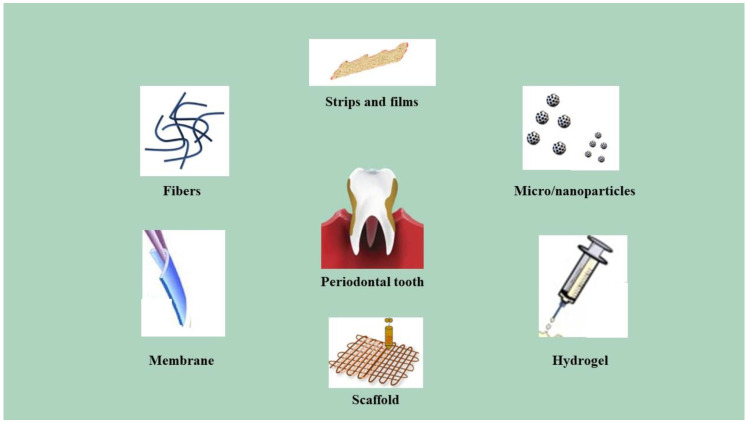
The available LDDSs for periodontal treatment.

LDDSs manage the release of locally administrated drugs that are indicated as an adjunct to periodontal treatment. There are two groups: LDDSs loaded with therapeutical agents used as an adjunct in non-surgical periodontal therapy, and LDDSs loaded with drugs used as an adjunct in surgical periodontal therapy (Table 1). Local administration is suitable to treat periodontitis since systemic administration is usually related to gastrointestinal issues, the need for frequent doses to maintain high blood concentration of the drug, and other collateral effects, such as dysbacteriosis or drug resistance [31]. Since the drug used to prevent and treat periodontitis needs to stay in the periodontal pocket for a sufficient timelapse at a high enough concentration, LDDSs have shown to be necessary [48,49,50]. They are introduced directly into the periodontal pockets to provide control of the SRP-adjuvant drug used. Locally administrated drugs do not undergo typical systemic drug-associated problems, but the role of gingival crevicular flow (GCF) should be analyzed; it may influence the outcome of the local administrated drug. GCF flow (or flow rate) indicates the process of gingival crevicular fluid moving in and out of the pocket area. The GCF flow is composed of the following three components: the resting volume, the influx, and the efflux. Due to the fact that the resting volume is constant over the measurement period and that it has minimal losses caused by evaporation or absorption, GCF flow can be measured by considering either the influx or efflux. It has been demonstrated that the GCF flow increases in periodontitis-affected sites. For this reason, it has been suggested that it could become a parameter to evaluate therapeutical responses, hypothesizing that its new measure would be close the GCF flow measured at healthy sites as an effect of the locally administrated drug. In a study [51], this hypothesis was tested by the introduction of tetracycline-loaded fibers. It decreased, however, since the changes of GCF flow rate were too variable, and it was assessed that it could not be considered statistically relevant as changes in PD and CAL to evaluate the efficiency of the adopted therapy [52]. Moreover, an LDDS for periodontal treatment are advantageous compared with the systemic administration of drugs because of the following features: it guarantees minimal invasiveness and the direct administration of the drug in the interested situ; thus, it acts immediately in the affected site and it has contact with that site only, without having to wait for the process of transport of such molecule and without compromising other organs. It bypasses the hepatic metabolism; thus, there is not percentage of the drug that goes lost, as in the systemically administrated ones. It avoids gastrointestinal issues, which is typical of orally administrated drugs, as they often irritate the stomach mucosa or they cause dysbacteriosis of the intestinal tract if they are antibiotic, leading to diarrhea as a consequence. It is associated with the reduction in the frequency of the doses because, as said before, 100% of the molecule is effective since no part of it is metabolized by the liver. It increases the compliance of the patient due to the fact that is very easy to administer, and it gives the possibility to introduce drugs that are not compatible with systemic administration, such as chlorhexidine [32,46]. LDDSs do not lack some disadvantages. In fact, some local drug delivery systems have difficulties in management, and some of them have difficulty providing sufficient drug concentrations. The oldest examples are not biodegradable, and they are associated with the discomfort of a second intervention to remove them. They are relatively new; thus, there is still the need of further investigations to assess which kind of LDDS is the best one. Last, but not least, local drug delivery systems are often associated with high costs, which may hamper their clinical appliances [31,47] (Table 2). The ideal features of LDDSs are to be biodegradable, biocompatible, easily administrable, to release the drug in a controlled way, to maintain its concentration as stable for a long time, and to not be irritative [32]. We have the following LDDSs available: fibers, strips and films, microparticles, nanosystems, gel, membranes, and scaffolds [31,32,53] (Figure 1) that can be associated with three types of drugs: anti-bacterial drugs, inflammation modulators, and alveolar bone and tissue repairing agents [31] (Table 1). LDDSs and their indications and contraindications are discussed in Table 3.

## 5. Types of LDDSs in Periodontitis Treatment

### 5.1. Fibers

**Figure 2:**
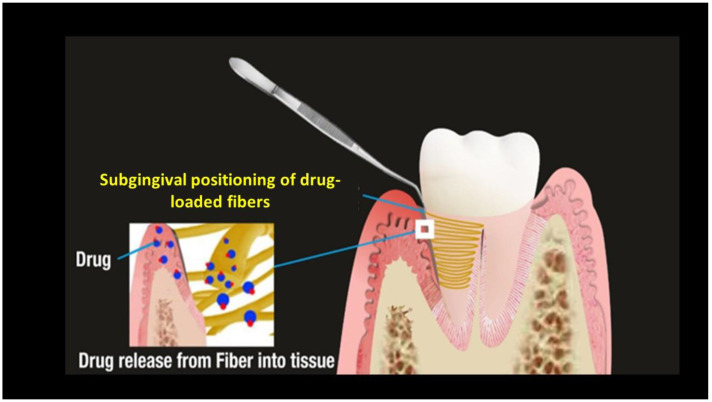
Fibers and their placement. Reproduced with the permission from Rajeshwari et al. [32].

Fibers are a reservoir-type delivery system loaded with the selected therapeutic agent, placed circumferentially into the periodontal pocket by an applicator and maintained in situ by a cyanoacrylate adhesive or a periodontal dressing [32,54,55] (Figure 2). A variety of polymers have been proposed and studied as fibers for local drug delivery systems: either natural, such as chitosan, zein, and gelatin, or synthetic, including poly(e-caprolactone), polyurethane, polypropylene, cellulose acetate propionate, and ethyl vinyl acetate [31,32]. All of them, when used and tested, were loaded with anti-bacterial drugs.

In 1979, thanks to Goodson et al. [47], hollow fibers impregnated with tetracyclines were proposed. The local administration of tetracyclines loaded in hallow fibers allowed the introduction of less than 1/1000 of the normal amount of tetracyclines by systemic administration. Nevertheless, the limitation of this formula was the duration of the concentration in the long term. For this reason, over the years, most scientists have tried to propose other kinds of fibers that would have avoided the aforementioned limit, such as Tonetti et al. [56] (Table 4), who made an ethyl vinyl acetate fiber loaded with 25% tetracycline that maintained constant levels of drug for 10 days.

**Figure 3:**
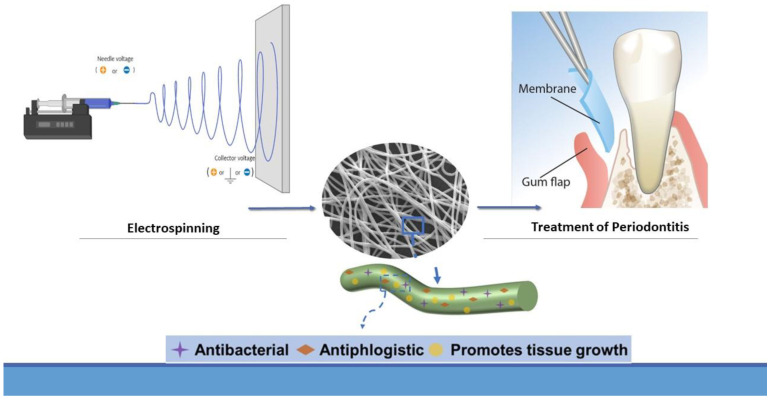
The procedure of electrospinning to make nanofibers. Each nanofiber has antibacterial and antiphlogistic activity and promotes tissue growth. Nanofibers are assembled in membranes and then applied to the periodontitis-affected site.

The first type of fibers was non-biodegradable, for this reason, it was associated with discomfort because it was necessary for a second intervention to remove them, and wound healing was associated with redness of gums [31]. To avoid this, biodegradable fibers were introduced in the market, including, for instance, collagen fibers [3]. It is important to highlight the new introduction of an interesting procedure to obtain polymeric nanofibers with superior biochemical properties, called electrospinning [12] (Figure 3). This procedure is used to make fibrous structures such as a native extracellular matrix, which can be functionalized to carry inorganic substances, bioactive factors, or chemical drugs [13]. The materials that have suitable features to be processed with electrospinning are poly lactic-co-glycolic acid (PLGA) and gelatin (GEL). In fact, they are extremely biocompatible and biodegradable [57,58].

In 2009, a study [3] that involved patients treated with SRP only and the test group of patients treated with SRP was associated with resorbable fibers of collagen loaded with tetracyclines. The positive impact of the use of fibers loaded with antibacterial drugs was assessed, and, in fact, the test group was associated with better results in terms of the reduction of probing depths (Table 1). Over the years, other studies, such as the randomized clinical trial of Chhina et al. [59], assessed by clinical and biochemical measurements that patients suffering from chronic periodontitis treated with SRP in combination with tetracyclines fibers had better outcomes than with SRP alone. However, that does not mean that SRP only is a bad therapeutical option. In fact, it is efficient for early-to-moderate periodontitis. Meanwhile, the association of SRP with LDDS fibers loaded with tetracyclines is a good option for the severe form of the disease.

In addition to the clinical aspects, in vitro studies about the use of fibers with anti-bacterial agents were conducted too. Studies in vitro evaluated the kinetics of the LDDS used since it is important to have a constant release of the drug at bacteriostatic or bactericidal concentrations, and the effect on the specific periodontal pathogenic microbes was also evaluated. Vijayalashmi et al. [4] (Table 1) tested tetracyclines-loaded collagen fibers in water and in a reproduced periodontal pathological pocket environment in serum, evaluating the kinetics of the drug and the effect on *P. gingivalis*. The results showed that this type of fiber released a controlled quantity of the drug for a therapeutical period (10 days) and was effective against *P. gingivalis* in the simulated periodontal pathological environment. Moreover, it was assessed that the increased release of the drug was associated with increased degradation of the fibers, and this would have a clinical impact since the amount of drug released and the, consequently, anti-microbial action would be deductible by just seeing the grade of degradation of the fiber.

Even the first studies and clinical applications of fibers were using tetracyclines. It has been demonstrated that other anti-microbial molecules, including metronidazole or azithromycin, are efficient too, giving better results than SRP alone in periodontal treatment [60,61]. Recently, other kinds of drugs have been tested because of the drug resistance associated with the long-term use of antibiotics [14]. For example, Ze He et al. [5] (Table 1) tested PLGA nanofibers loaded with tea polyphenols (TP), which are active compounds present in tea and are mainly composed of catechins and their derivatives, which have shown good influences on periodontitis treatment [15,62]. It has been demonstrated that TP plays an important role in fighting periodontal inflammation and in preventing it by decreasing the levels of interleukin-1(IL-1β) and tumor necrosis factor (TNF)-ɑ, which are the major pro-inflammatory cytokines relevant to periodontal destruction [62,63,64,65]. In this study, TP was part of the shell layer; meanwhile, the core layer was impregned with Adiporon (APR), an adiponectin mimetic that specifically binds to the adiponectin receptor and promotes alveolar bone regeneration by enhancing osteogenic differentiation [66]. The study’s results, conducted in vivo in mice and in vitro, gave great hopes. In fact, TP released in the early stages fought the inflammation, and ARP, released in later stages, promoted bone regeneration; both were present at effective concentrations.

In conclusion, fibers are one of the most ancient forms of LDDSs. They are suitable for inaccessible areas, but, if non-biodegradable fibers are used, they need to be removed after treatment, leading to gingival redness. Moreover, new directions are addressed to progressively tinier forms, such as nanofibers, and other drugs used than antibiotics, such as TP.

### 5.2. Strips and Films

**Figure 4:**
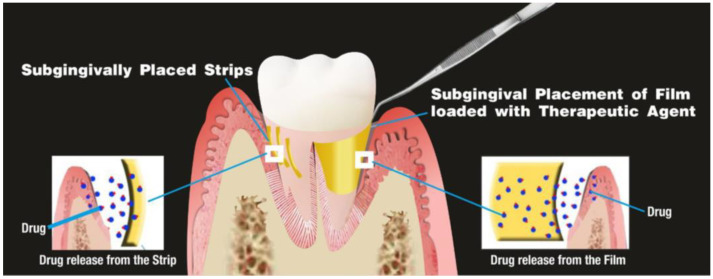
Strips and Films and their placement. Reproduced with the permission from Rajeshwari et al. [32].

Strips and films (SFs) are thin matrix bands in which drugs are dissolved throughout the polymer. SFs are great in matching the shape and size of the periodontal pocket and is, consequently, easy to insert with minimal discomfort for patients; they are placed in the interproximal periodontal pocket space (Figure 4) [32].

The first materials proposed in the fabrication of strips and films were acrylics loaded with different kind of antibiotics, and they showed a significant drug release on the first day, with a subsequent sustained release over 4–5 days after placement [6] (Table 1). As they were non-biodegradable, they were associated with the disadvantage of a second intervention for the removal, which is a difficult procedure because they soften in the crevicular fluid, and all this causes irritation in gums [67]. To overcome such a disadvantage, new bioabsorbable materials were introduced, including poly-hydroxybutyric acid and poly lactic-co-glycolic acid (PLGA), atelocollagen, gelatin, chitosan/PLGA, and more. They were tested and showed good results [31]. Non-biodegradable SFs released the therapeutical agent by diffusion. Meanwhile, biodegradable SF released by diffusion and erosion [68].

SFs loaded with antibiotics and antiseptic drugs have been studied, showing good long-term concentration maintenance and good clinical improvements of gingival health [7,69,70,71,72] (Table 1). In 2002, Friesen et al. [69] assessed the superiority of SRP associated with strips loaded with tetracyclines over SRP alone and demonstrated the major efficacy of multiple strips over a single strip in reducing probing depths. Even though chlorhexidine-loaded strips have been previously associated with the least efficacy in periodontal treatment when compared with other molecules [73], Paolantonio et al. [74] showed a significantly higher reduction of probing depths (*p* < 0.01) in sites treated with chlorhexidine chips than in sites treated with SRP alone.

In addition to the studies that involved antibiotics and chlorhexidine, herbal-derived agents have been introduced to avoid a major disadvantage associated with antibacterial drugs, i.e., antibiotic resistance. For example, Kudva et al. [75] studied the effect of green tea in periodontal health, demonstrating its bactericidal action that led to a better clinical aspect of gums.

Finally, SFs have the same range of materials used to make them as the fibers. They differ in their release rate, according to their dimensions and also in their application. In fact, fibers are suitable for inaccessible and the most distal regions. Meanwhile, SFs are wider, and, for this reason, they are suitable for larger pocket areas [16]. Nowadays, the market has introduced smaller LDDSs, such as nanoparticles, microspheres, and gels, stealing the scene from fibers and SFs.

### 5.3. Microparticles

**Figure 5:**
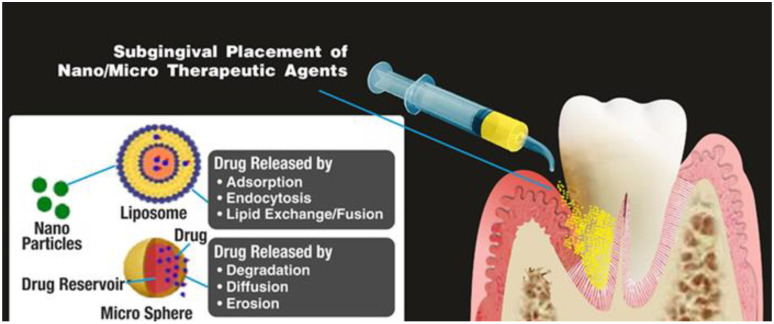
Microparticles and nanosystems and their placement, reproduced with permission from Rajeshwari et al. [32].

Microparticles are solid spherical polymer structures with a diameter from 1 to 1000 μm, loaded with a drug that spreads uniformly throughout the polymer matrix. They are very easy to administer and provide a prolonged release of the drug but are not readily retained in the targeted site. They are delivered via various carrier systems such as chips, dental pastes/gel systems, and direct injection into the pocket [32] (Figure 5).

Materials of natural origins, modified natural substances, and synthetic polymers, divided into biodegradable and non-biodegradable categories, have been proposed for microparticles [31]. In 1997, Esposito et al. [17] tested in vitro three different types of microparticles (poly(L-lactide), [L-PLA] poly(DL-lactide), [DL-PLA], and poly (DL-lactide-co-glycolide) 50:50, the [DL-PLG]) loaded with tetracyclines. They showed differences in the release kinetics related to the material that the microparticles were made of. In addition, all of them released tetracyclines in a controlled manner for two weeks, making them promising clinical applications. Usually, tetracyclines-loaded (lactic-co-glycolic acid) (PLGA) microparticles are used, but they are associated with the following disadvantages: low loading efficiency for encapsulating highly water-soluble drugs into PLGA microspheres/nanospheres [76,77]; and the slow degradation rate of PLGA, which causes the presence of empty microspheres/depots at the site of periodontal pockets for a long time after the loaded minocycline is completely released [78]. To avoid such limits, scientists are constantly looking for the best formulation of LDDSs to achieve the maximum yield. Recently, ion pairing and complexation has been introduced as a new promising strategy for local drug delivery and release [79,80] since it could enhance the therapeutic performance of active pharmaceutical components by altering their solubility, stability, release rates, and bioactivity [76,81,82]. Wu et al. [8] (Table 1) investigated the possibility of exploiting the ion pairing/complexation of minocycline, Ca2+, and sulfate/sulfonate-bearing biopolymers to develop an intrapocket delivery system of minocycline as an adjunct to scaling and root planing. The study was conducted in vitro, and a high loading efficacy (96.98% ± 0.12%) was observed with a high loading content (44.69% ± 0.03%) in minocyclines for these complex microparticles, compared to the usual loading content that does not go over 10%. Moreover, thanks to agar disk diffusion and biofilm assays, the antimicrobial effect against *Streptococcus mutans* and *Aggregatibacter actinomycetemcomitans* was assessed. These results gave great hopes for the clinical application of microparticles made by ion pairing and complexation.

In the research of the best formulation of microparticles, the evaluation of cross-linked chitosan microparticles [54] containing metronidazole (MTZ) by Pichayakorn et al. [9] (Table 1) is worth mentioning. In this in vitro study, it was revealed that the best formulation among the tested ones was MTZ-MPs with the following variables: composed of 1% of Span80 in soybean oil, 5% glutaraldehyde in chitosan solution, 30 min of cross-linking time, 1:1 drug/chitosan ratio, and drug adding in the form of ethanol solution and washing with hexane only. The MTZ-MPs had a 59.40% entrapment efficacy and a prolonged release profile. In the same study, another evaluation was made: the drug release of the MTZ-MPs in hydrogels and in films compared to drug powders. This analysis was made based on a previous study [83], in which both chitosan-based hydrogels and films have been recognized to have excellent features for local drug delivery in the oral cavity, including mucoadhesiveness, biocompatibility, and biodegradability. The results demonstrated the superiority of MTZ-MPs hydrogels and MTZ-MPs films over powders providing prolonged release, and, in particular, MTZ-MPs hydrogels provided constant release rates during the studied period of 6 h, while MTZ-MPs films provided a fast release in the initial 5 min and then reached a plateau.

In addition to in vitro studies, clinical investigations of the application of microparticles as LDDSs in periodontitis treatment have been conducted too. The application of solid lipid microparticles (SLMs) is interesting in the study of Gad et al. [84]. SLMs have acquired particular concern in medicine thanks to the following properties: its physiologically well-tolerated nature, high biocompatibility, avoidance of organic solvents, ability to modify and target drug release, increased drug stability, high drug load, and ability of large-scale production by a high-pressure homogenization technique [85]. Gad et al. [84] developed SLMs encapsulating doxycycline hydrochloride DH and metronidazole MTZ via the hot homogenization method. The type of lipid matrix, homogenization speed, drug amount, surfactant type, and concentration influenced the % of entrapment efficacy, particle dimension, and zeta potential of the SLMs. The optimal formulation of the SLMs was made of 5% *w*/*w* TP as a lipid matrix, with 25-mg DH and 100-mg MTZ, which was then stabilized with 5% *w*/*w* P188 + EP. It was subjected to freeze-drying and showed adequate stability when stored at both ambient and refrigeration temperatures. Then, SLMs were incorporated into poloxamer gel, which slowed down the rate of release of DH and MTZ and gave a satisfactory local drug-delivery system. In conclusion, the adjunctive use of SLM gel to a conventional SRP resulted in a significant decrease in the total amount of anaerobic bacteria from pre-therapy to post-therapy and in good clinical results of periodontal parameters in patients affected by chronic periodontal disease.

In sum, microparticles have been extensively studied, and new formulations are proposed every day to improve their activity and efficacy. It would be interesting to find out what will happen when the formulations tested in vitro will be tested in humans. By comparing the results of studies in humans, it will be clearer what formulation best fits ordinary clinical practice and should be used by most clinicians.

### 5.4. Nanosystems

Nanosystems are characterized to have very small sizes that allow them to be suitable for the areas where other forms of LDDSs do not arrive, such as the pocket area below the gum line. They are directly injected in the pocket area or placed via other carrier systems (ex. gels) [32] (Figure 5). They include micellas, metallic and polymeric nanoparticles, liposomes, and nanofibers. They are optimal because they have high loading capacity and a favorable surface–volume rate. Some of them have also shown antibacterial properties, which could be an advantage in treating periodontitis since it is caused by bacterial etiological factors. In particular, metallic nanoparticles are used in dentistry for their antimicrobial activity and bone regeneration properties [19,31,32,86], but they are, unfortunately, associated with citotoxicity and non-degradability, making them unsuitable to be used for periodontal therapy [87,88]; hence, other materials are preferable to be used. Polymeric nanoparticles loaded with minocycline by emulsification–diffusion have shown good results, having 96% of the minocycline released after 12 days and very good clinical outcomes in terms of periodontal healing [89]. Chitosan, which is a natural material, characterized by high biodegradability, nontoxicity, and antimicrobial properties [90], has been largely used in the fabrication of nanoparticles. For example, Xu et al. [10] (Table 1) made doxycycline-loaded chitosan nanoparticles with a mean particle size of 50 nm, which showed an around 75% entrapment efficiency and a 28% loading power. The results revealed that the preparation presented good cell compatibility, better antibacterial properties against *P. gingivalis*, and successful downregulation of inflammatory factors.

One of the best biocompatible and biodegradable materials is PLGA. PLGA nanoparticles have been largely investigated in medicine, even in neurobiology [82]. PLGA loaded with doxycycline (DOX) has shown a sustained release after administration in periodontal pockets, a good adjunctive therapy to SRP. Moura et al. [91] investigated DOX-loaded PLGA in a clinical trial, collecting samples of gingival crevicular fluid (GCF) 2, 5, 7, 10, 15, and 20 days after DOX administration. The significant decrease in drug concentration in GCF was observed only on day 20 (19.69 ± 4.70 µg/mL). Meanwhile, until day 7, DOX concentration was stably sustained (23.33 ± 1.38; 23.4 ± 1.82; 22.75 ± 1.33 µg/mL, respectively), and it started to decrease at days 10 (21.74 ± 0.91 µg/mL) and 15 (20.53 ± 4.88 µg/mL).

PLGA nanoparticles have also been tested clinically in combination with antimicrobial photodynamic therapy (aPDT). The addition of aPDT to SRP has been largely investigated and has resulted in being controversial since it has never provided significant benefits unless applied in multiple sessions [18,92,93,94]. De Freitas et al. [95] tested the synergetic effect of aPDT with Methylene Blue (MB)-loaded PLGA nanoparticles on human dental biofilm microorganisms in vitro (planktonic and biofilm phase) and in vivo (patients with chronic periodontitis). The choice to test the above-mentioned combination takes advantage of nanoparticles’ high penetration and sustained drug release properties [96,97] that could have enhanced the antibacterial activity of aPDT. In biofilms, MB nanoparticles killed 25% more bacteria than free MB. In patients, the clinical safety of aPDT was assessed, and, at first, both the test and the control group showed similar results, then, after three months, ultrasonic SRP + aPDT (test group) showed a greater effect (28.82%) on the gingival bleeding index (GBI) compared to ultrasonic SRP (control group).

Since a new tendency is to avoid the use of antibiotics as much as possible, such as for aPDT, other therapies are being tested. For example, very recently, Perez-Pacheco et al. [98] investigated the possibility of using nanoparticles loaded with curcumin since it has been demonstrated that it has anti-microbial, anti-carcinogenic, antiviral, antioxidant, and anti-inflammatory properties [20,99,100,101]. They tested the efficacy of a single local administration of 0.05 mg/mL nano-encapsulated curcumin and assessed that it does not provide significant benefits to SPR; thus, it is unnecessary to use it as adjunctive therapy in periodontal treatment. In contrast, one year later, some of the same scientists of the aforementioned study investigated the role of curcumin-loaded nanoparticles in the experimental periodontal model in rats and discovered that it promoted bone regeneration [102]. For this reason, the potential use of curcumin as an adjunctive therapy for periodontal disease requires further investigation.

The increasing importance of liposomes as a carrier for controlling periodontal inflammation in patients is worth highlighting. In fact, liposomes can improve drug uptake into cells, change drug pharmacokinetics and biodistribution, and release an encapsulated drug in a controlled manner [103]. On the other hand, another type of drug that has shown good results in the treatment of periodontitis by local administration is represented by statins [21].

In conclusion, many types of loaded drugs to formulate are available and are promising to be used in nanocarriers. Despite the numerous possibilities nanoparticles have, they also have some disadvantages. In fact, they are expensive, their production process is very complicated, and their formulations lack stability [31].

### 5.5. Gels

**Figure 6:**
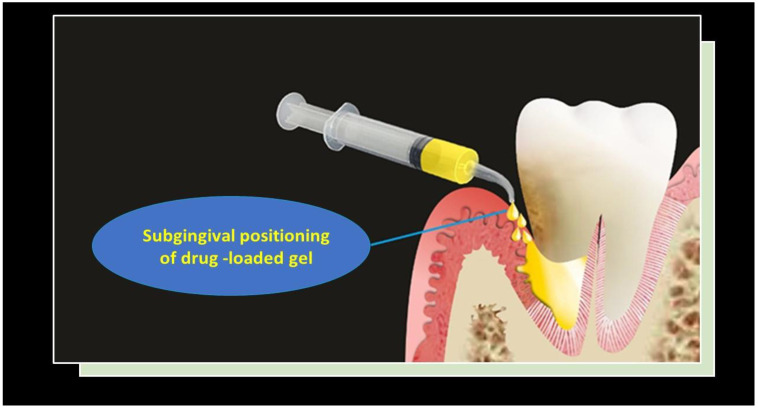
Gels and their placement. Reproduced with the permission from Rajeshwari et al. [32].

Gels are very popular in dentistry, thanks to their multiple advantages: they have high biocompatibility and bioadhesivity, very easy administration, and easy fabrication. They are placed by wide-port needle syringes in the periodontal site [32] (Figure 6).

Various types of polymers, such as carbopol, carboxy methyl cellulose, and chitosan, can make them. In particular, chitosan is a material widely used in dentistry, especially for the periodontal cure. In fact, it was previously mentioned in other formulations of local drug delivery systems. It is used thanks to its biological properties, such as its antibacterial, anti-inflammatory, and wound healing properties [104]. It has been applied as a gel, giving good clinical outcomes [105] and being effective against periodontal pathogens, such as *Porphyromonas gingivalis* [106].

Nowadays, the recent innovation of gel formulations is represented by in situ formed gels that go through liquid-to-semisolid state transitions as a reaction to stimuli such as temperature changes or solvent effects [79,107,108]. Even though gels are good delivery systems, they have been associated with a big disadvantage: a relatively rapid release of the captured drug. For this reason, researchers have developed formulations of a combination of gels and other drug delivery systems. Wang et al. [109] designed, fabricated, and tested in vitro and in vivo in rats a tunable and injectable local delivery system by loading the poly (lactic-co-glycolic acid) (PLGA)-drug microspheres into thermo-reversible polyisocyanopeptide (PIC) hydrogel. New thermo-reversible PIC hydrogels have been demonstrated to be easier to penetrate deep and irregular pockets, where gelation will take place immediately in situ upon reaching body temperature, especially when compared to the current market-available gel formulations that are more viscous and not freely flowable (Periocline^®^—Sunstar, Inc. (Osaka, Japan) and ATRIDOX^®^—Tolmar GmbH, Fort Collins, CO, USA). In this formulation, they incorporated doxycycline and lipoxin, which were separately loaded into acid-terminated and ester-capped PLGAs by electrospraying. The results showed appropriate injectability, long-term structural stability, and no evident in vivo inflammatory response. It is important to highlight that the stability test was conducted in vitro; thus, its limit is that it should be investigated in vivo in humans to have more accurate results since the periodontal pocket is subjected to various forces, mostly related to mastication. An interesting fact that emerged from this study is that the drug release could be manipulated from 1 week to 6 weeks by correcting the loaded mass ratio of acid- and ester-end capped PLGA microspheres.

Mou et al. [11] (Table 1) developed serum albumin microspheres containing minocycline and zinc oxide nanoparticles (ZnO NPs) in a Carbopol 940^®^ hydrogel. This study gave promising results. In fact, by the in vitro test, its great encapsulation efficiency (99.99%) for minocycline was assessed, and the slow-release time was more than 72 h with pH-sensitive properties. Moreover, an in vitro test was conducted to evaluate the safety of this formulation towards cells. Cell survival rates were over 85% below 0.8 mg/L of ZnO NPs, assessing low toxicity and high security that allowed future clinical tests to introduce this promising formulation in the market as a recognized adjunctive periodontal therapy.

Very recently, another experimental study was conducted in vitro and in vivo in rats. Lu et al. [110] developed I2@COF-HEC hydrogel, using the cross-linked cyclodextrin metal-organic framework (COF) as a carrier for iodine, and further suspended it in hydroxyethyl cellulose gel. They assessed the sustained release of the formula in artificial saliva and evaluated in vivo rat periodontitis models’ capacities to inhibit bone resorption and alleviate periodontal inflammation. These results hope to further the experiments of this formula in humans to assess its clinical safety and efficacy.

In addition to the in vitro promising studies, others have conducted clinical trials. For example, as already mentioned in the section of nanosystems, Gad et al. [84] formulated solid lipid microparticles (SLMs) gels encapsulating doxycycline hydrochloride (DH) and metronidazole [111] and demonstrated the clinical efficacy of the formulation in periodontal patients.

Furthermore, we have discussed the various formulations that are mostly loaded with antibiotics, but, as said before, it is a challenge to substitute antibiotics with other therapeutical agents that can provide the same benefits without causing bacterial resistance. Among the therapeutic agents that are already tested and currently applied in LDDSs as gels, we have anti-inflammatory drugs [22,23,24,112,113]. Their application has been demonstrated to produce effective benefits in periodontal health and sustained drug release; for example, Xu et al. [25] produced and tested an injectable and thermosensitive gel loaded with aspirin and erythropoietin, which had excellent biocompatibility, was easily prepared, and could continuously release the active substance for at least 21 days. Moreover, there are interesting studies about gels loaded with encapsulated osteogenesis drugs [26,27]. For instance, the biological hydrogel of recombinant human Fibroblast Growth Factor type 2 (rhFGF-2) in a hyaluronic acid (HA) has been tested, showing more PD reduction (5.5 versus 2.9 mm), PAL gains (4.8 versus 2.2 mm), and shallower residual PD (4.2 versus 6.6 mm) than controls [114].

Among the newly tested molecules, there are natural agents. One of the most recent studies is the one by Qi et al. [115], in which, based on the principle of oxidative self-polymerization, Turkish Galls effective constituent (TGEC, T) was loaded into nanoparticles (T-NPs). T-NPs were compressed into thermosensitive in situ hydrogel, and, from them, 42.29 ± 1.12% of TGEC, T was constantly liberated in 96 h under periodontitis conditions. The effects were that T-NPs caused the lysis of bacteria. In fact, they favored the enormous production of ROS without damaging the periodontal tissue. It is only the beginning, but these results are promising for further investigations to find a suitable substitute drug for antibiotics. Another interesting new study assessed the added benefit of 4% mangosteen gel to SRP [28], demonstrating once more the efficacy of natural agents that represent future therapeutical chances.

In conclusion, gels are very popular in periodontal treatment, and a lot of formulations of gels/hydrogels alone or in combination with other LDDS are being produced and tested. They seem to be one of the best therapeutical options, not just for good results in terms of encapsulation efficacy, biodegradability, biocompatibility, stability, and drug release but also because they are more comfortable and acceptable for the patients.

### 5.6. Membranes

**Figure 7:**
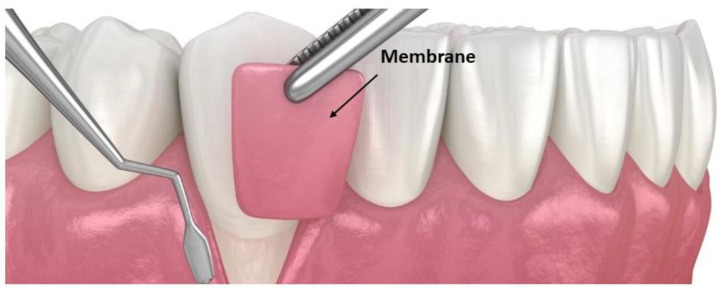
Membrane and its placement in order to act as a barrier.

In periodontitis, the advance of biofilm directed apically causes bone resorption. For this reason, it is important to provide bone regeneration in order to correct such bone defects. To make it possible, membranes have been developed and applied. They work as barriers promoting the wound healing of periodontal tissues (Figure 7) [29,30] and can be combined with some therapeutical agents that enhance such properties, making them LDDSs [116]. First, non-biodegradable membranes were introduced. Then, they have been progressively abandoned because of the need for a second surgical intervention to remove them [117].

Nowadays, absorbable membranes have supplanted old non-resorbable membranes, and they have a functional layer in which growth factors and osteogenesis drugs are contained. Current membranes used in periodontal regeneration and GTR are in collagen or polyglycolide and/or polylactide or their copolymers. Collagen is the most used material thanks to its advantages: it does not cause immune reactions; it engages and turns on gingival fibroblast cells and it has hemostatic power [118]. It has been demonstrated that collagen membranes promote fibroblast DNA synthesis, and, in addition, osteoblasts show more adherence to collagen membrane surfaces when compared to other membrane surfaces [119].

Recently a core–shell nanofibers membrane has been designed to treat periodontitis. Liu et al. [120] developed this membrane, loading an inhibitor (SP600125) in the polymeric micelles of the shell and the recombinant human bone morphogenetic protein-2 (BMP-2) in the core. The results were very promising. In fact, it detected good degradation performance and had a prolonged release profile of up to one month. Moreover, in the in vivo study, the nanofiber membrane inhibited alveolar destruction and recovered bone defects. These great outcomes make such a formulation a candidate to be a future therapeutical alternative to periodontitis treatment.

Even though the use of membranes as LDDS loaded with growth factors or other therapeutical drugs that induce bone regeneration has been discussed, antibiotic membranes have been tested too. Ho et al. [121] studied an antibiotic membrane made by PDLLA electrospun nanofibers loaded with amoxicillin (AMX). They tested its efficacy in vitro and in vivo in rats. They assessed that AMX had an 81.16% ± 10.51% encapsulation efficiency. AMX was freed in a constant way from the PDLLA nanofibers, following a biphasic pattern characterized by, first, a fast release and, second, a constant release over a period of 28 days. During the first week of membrane installation, approximately 60% of the AMX was liberated. The results of this study were promising; thus, this kind of formulation should be further investigated in order to apply it clinically. Even though this study is very interesting, especially concerning the fabrication of nanofibers and their capability of loading and releasing the drug, the choice of using antibiotics represents a step back compared to current pharmacological challenges. In fact, nowadays, there is the need to abandon the use of antibiotics as much as possible due to their drawbacks; thus, it would be much more appealing to test this formulation loaded with other therapeutical agents.

### 5.7. Scaffolds

**Figure 8:**
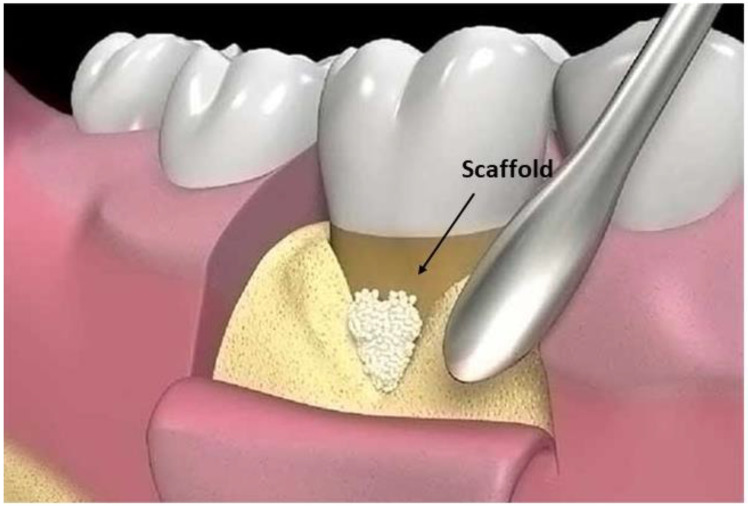
Scaffold and its placement.

To correct bone defects, scaffolds have been introduced for the same purpose as membranes. They are preferable since they avoid the main limit of absorbable membranes: the weakness that does not permit sufficient mechanical resistance to external forces. They are placed in the affected area to maintain the space for subsequent periodontal tissue regeneration [122] (Figure 8).

A tetracyclines-loaded chitosan scaffold has been tested, detecting a higher loading capacity when the percentage of chitosan and glutaraldehyde was higher [33]. Liao et al. [34] evaluated the antibacterial effects and controlled-release capacities, osteogenic and cementogenic effects in vitro, and a mesoporous hydroxyapatites/chitosan (mHA/CS) composite scaffold loaded with recombinant human 20 μg/mL amelogenin (rhAm) in vivo. The results demonstrated an inhibitory effect against *Fusobacterium nucleatum* and *Porphyromonas gingivalis*, promoting bone regeneration in vitro and cementum regeneration in vivo, giving hope for its future clinical application. An interesting discovery was the impact of the small molecule peptide galanin GAL on periodontal regeneration. Since periodontal disease was associated with the downregulation of GAL, the research group of Ma et al. [123] tested GAL-coated scaffolds for periodontal regeneration purposes in vitro and in vivo in rats. The results were promising, with good periodontal regeneration properties.

**Figure 9:**
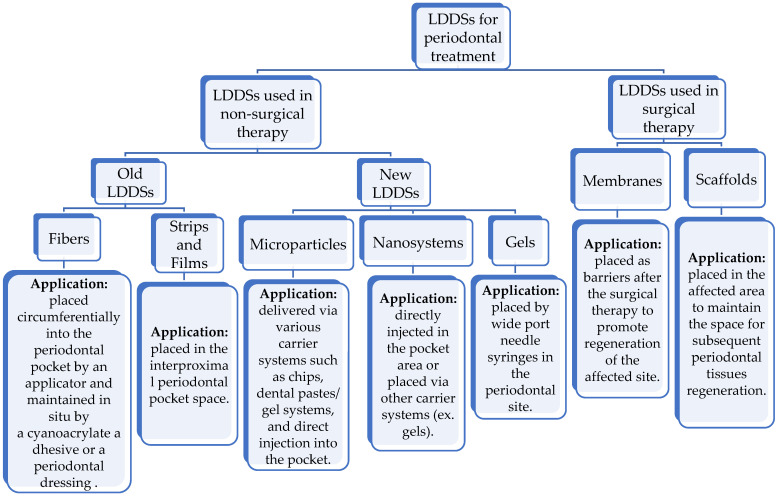
A flow chart of the classification of LDDSs and their modalities of application [32,122].

Stem cells are gaining importance in periodontal regeneration [35] and can be incorporated into the scaffold to promote their delivery. Baba et al. [36] demonstrated the benefits of the implantation of autologous mesenchymal stem cells (MSCs) with a biodegradable three-dimensional (3D) woven-fabric composite scaffold and platelet-rich plasma (PRP) in periodontal regeneration. They assessed the safety of this formulation, and good clinical outcomes were demonstrated by the improvement of the following three parameters: clinical attachment level, pocket depth, and linear bone growth (LBG). It is worth saying that future tendencies could be toward a scaffold-free approach, even if scaffolds are a good therapeutical option, using stem cells only or in combination with growth factors [124]. However, the scaffold-free approach is still difficult since it lacks sufficient data in humans [35,37] (Figure 9).

## 6. LDDSs and Systemic Diseases in Periodontal Patients

Periodontitis has been demonstrated to be associated with some systemic diseases and disorders, including diabetes mellitus, pregnancy complications, cardiovascular disease, metabolic disease and obesity, rheumatoid arthritis, certain cancers, respiratory diseases, and cognitive disorders, such as Alzheimer’s disease [125]. Diabetes has been shown to have a strong impact on periodontitis, leading to a higher incidence of periodontitis and degree of severity in diabetic patients [126,127]. We have already discussed the advantages that LDDS have compared to systemic administration. In addition, they are suitable for diabetic patients because they often have other associated systemic diseases and need to use several medicines, augmenting the risk of drug interactions if systemic administration was the only chance [128]. For this reason, LDDSs have been tested in periodontal diabetic patients. Lecio et al. [129] evaluated the clinical, microbiological, and immunological results of poly lactic-co-glycolic acid (PLGA) nanospheres containing 20% doxycycline (DOXY) in the treatment of type-2 diabetic patients (DM-2) with chronic periodontitis (CP). They assessed the efficacy of this formulation in addition to SRP. In fact, it was observed that the number of sites showing PD reduction and CAL gain ≥2 mm was higher 3 months later (*p* < 0.05) in the group of patients treated with DOXY. Moreover, the following changes were detected in the DOXY group: significant augmented levels of anti-inflammatory interleukin (IL)-10 and decreases in IL-8, IFN-y, IL-6, and IL-17 (*p* < 0.05), a significant decrease in periodontal bacteria (*p* < 0.05), and a lower mean percentage of HbA1C 3 months later (*p* < 0.05). Other formulations and loaded drugs have been tested too, but they were conducted in animals. There are two studies in periodontal diabetic rats, in which the benefits of metformin-loaded PLGA nanoparticles and silk fibroin nanoparticles loaded with resveratrol were assessed [130,131]. The results of these two studies allowed researchers to continue on with experiments on humans. This way, we will have a wider spectrum of therapies from which to choose the best one that fits diabetic patients.

For the other systemic diseases and disorders, there are not studies about the application of drugs in LDDS. Some studies have investigated the role of adjunctive drugs in periodontal rats affected by estrogen deficiency [132], hypertension [111], and rheumatoid arthritis [133], giving good promising results. Thus, the research should go further in the application of these drugs in humans, mostly using an LDDS to guarantee an optimal therapy for patients affected by systemic diseases.

## 7. New Perspectives

Very recently, Boese et al. [134] developed a drug-loaded coated floss for local drug delivery into periodontal pockets and studied its action in porcine jaws in ex vivo conditions. The first difference with the above-mentioned LDDSs is that floss had temporary contact with the interested tissues. In fact, it did not stay in the pocket, and it did not require a second visit to the dentist. The study used an un-waxed nylon braided floss, and it was dip-coated with model hydrophilic and hydrophobic drugs either in free form or after encapsulation in poly(lactic-co-glycolic acid) particles. They were able to coat it with up to 1.6 mg of particles. Coated floss was passed within the gum pocket of the excised porcine mandibles three times, and delivery efficiency was demonstrated to be up to 91%. Thus, this study gives great hope in using a minimally invasive and very easy-to-use kind of LDDS, even though it is just the beginning. In fact, new studies in vitro and in humans should be executed to assess their clinical applications. In addition to the drug-loaded coated floss, new directions concerning the analyzed LDDSs should be researched to determine the best formulation. We have discussed the combination of microparticles or nanosystems with gel, in which each part enhanced the effects of the other part. Moreover, finding out which is the best drug to load, in substitution for antibiotics, is challenging. Natural agents have shown promising results that require further investigation.

## 8. Conclusions

In conclusion, we believe that many formulations, even if promising, need further studies in humans to have enough data about the advantages and disadvantages of each one in clinical practice. The best formulation should provide short treatments, eliminate pain, ascertain faster patient recovery, and be comfortable, minimally invasive, biocompatible, and affordable. For this reason, we believe that the LDDS that could be the most promising is a gel alone or in combination with the presented LDDSs in this review. It has the aforementioned ideal features and would be very well accepted by the patients. On the other hand, antibiotics are the most used drugs for LDDS, but they have the following disadvantages: gastrointestinal collateral effects and bacterial resistance. This is why other therapeutical agents are gaining importance, including anti-inflammatory drugs, such as aspirin and erythropoietin, and natural agents, such as Tea polyphenols (TP), mangosteen, and Turkish Galls effective constituent (TGEC). All of them have been tested and have given promising outcomes that candidate them as alternatives for antibiotics. Finally, future research should focus on gels loaded with autoinflammatory drugs or natural agents, avoiding the collateral effects of antibiotics.

## Figures and Tables

**Table 1:** Stages of Periodontitis with associated therapy and the studied adjunctive therapeutical agents with relative LDDSs.

Periodontitis	Therapy	Studied Adjunctive Therapeutical Agents	LDDS for Adjunctive Therapeutic Agents
Stage I, II, III	SRP, motivation and education for domiciliary oral hygiene, correction of bad habits (ex. Smoking), control of systemic diseases (ex. Diabetes). [1]	Anti-bacterial and anti-septic drugs (tetracyclines, metronidazole, azithromycin, metronidazole, chlorhexidine), inflammation modulators (statins, lipoxin, aspirin, erythropoietin), natural agents (TP, curcumin, TGEC, mangosteen) [3,4,5,6,7,8,9,10,11,12,13,14,15,16,17,18,19,20,21,22,23,24,25,26,27,28,29,30]	Fibers, Strips and Films, Microparticles, Nanosystems, Gels. [31,32]
Stage IV	SRP, motivation and education for domiciliary oral hygiene, correction of bad habits (ex. Smoking), control of systemic diseases (ex. Diabetes), surgical correction of bone defects. [2]	Antibiotic (amoxicillin), alveolar bone and tissue repairing agents (inhibitor SP600125, BMP-2, rhAm, GAL, MSCs) [33,34,35,36,37]	Membranes, Scaffolds. [31]

**Table 2:** Advantages and disadvantages of systemic and LDDS administrations.

Type of Administration	Advantages	Disadvantages	References
Systemic	Some patients may prefer conventional drug- administration; well-known associated risk; no need of second intervention; cheap.	Low bioavailability of the drug; need for frequent doses; gastrointestinal issues; dysbacteriosis; drug resistance; interaction with other systemic administrated drugs.	[31,45]
LDDS	High bioavailability of the drug; controlled drug release; bypass of the hepatic metabolism; no gastrointestinal issues; reduction in frequent doses; mini-invasiveness of some LDDSs; high compliance of the patient; use of drugs that are not compatible with systemic administration (ex. Chlorhexidine); no interaction with other drugs.	Difficulty of management of some types of LDDS, some of them have difficulty to provide sufficient drug-concentration; the need of reintervention for the oldest LDDS; the need of further investigations to assess which kind of LDDS is the best one; high costs.	[31,32,46,47]

**Table 3:** Indications and contraindications of each LDDS [16,31].

Type of LDDS	Indications	Contraindications
Fibers	Adjunct to SRP, suitable for inaccessible areas	Larger pockets areas, surgical correction of periodontal site
Strips and Films	Adjunct to SRP, suitable for larger pocket areas	Inaccessible posterior sites, surgical correction of periodontal site
Microparticles	Adjunct to SRP	surgical correction of periodontal site
Nanosystems	Adjunct to SRP	surgical correction of periodontal site
Gels	Adjunct to SRP	surgical correction of periodontal site
Membranes	Adjunct to surgical correction of periodontal site	Not indicated as adjunct to periodontal therapy if it does not require surgical correction of the bone defect
Scaffolds	Adjunct to surgical correction of periodontal site	Not indicated as adjunct to periodontal therapy if it does not require surgical correction of the bone defect

**Table 4:** A summary of the characteristics of some of the studies that are reported in the review.

System	Material	Drug	Study Design	Time of Constant Drug Release	% Drug Loaded	% EE	References
Fibers	Ethyl vinyl acetate	Tetracyclines	Clinical trial	10 days	25%	Not mentioned	[56]
Fibers	Collagen	Tetracyclines	Case control	Not mentioned	Not mentioned	Not mentioned	[3]
Fibers	Fibrillar collagen	Tetracyclines	In vitro	10 days	Not mentioned	Not mentioned	[4]
Nanofibers	PLGA	TP	In vitro/in vivo	14 days	Not mentioned	Not mentioned	[5]
Strip	Polyhydroxybutyric acid	Tetracycline hydrochloride or metronidazole	In vitro/in vivo	4/5 days	25% metronidazole, 25% tetracycline HCI, 10% tetracycline HCI	Not mentioned	[6]
Film	Hydroxypropylcellulose	Chlorhexidine diacetate	In vitro	3 days	20% chlorhexidine	Not mentioned	[7]
Microparticles	Sulfate/sulfonate-bearing biopolymers	Minocycline	In vitro	9 days at pH 7.4 and 18 days at pH 6.4	44.69% ± 0.03%	96.98% ± 0.12%	[8]
Microparticles	Cross-linked chitosan	Metronidazole	In vitro	Prolonged release (not specified how many days)	Not mentioned	59.40%	[9]
Nanoparticles	Chitosan	Doxycycline	In vitro	Not specified	28%	75%	[10]
Microspheres in combination with Hydrogel	serum albumin microspheres containing minocycline and zinc oxide nanoparticles (ZnO NPs) in a Carbopol 940^®^ hydrogel.	Minocycline	In vitro	slow-release time was more than 72 h with pH-sensitive property	Not mentioned	99.99%	[11]

## Data Availability

Data are available from the corresponding author upon reasonable request.

## References

[1] Sanz M., Herrera D., Kebschull M., Chapple I., Jepsen S., Beglundh T., Sculean A., Tonetti M.S. (2020). Treatment of stage I-III periodontitis-The EFP S3 level clinical practice guideline. J. Clin. Periodontol..

[2] Herrera D., Sanz M., Kebschull M., Jepsen S., Sculean A., Berglundh T., Papapanou P.N., Chapple I., Tonetti M.S. (2022). Treatment of stage IV periodontitis: The EFP S3 level clinical practice guideline. J. Clin. Periodontol..

[3] Panwar M., Gupta S.H. (2009). Local Drug Delivery with Tetracycline Fiber: An Alternative to Surgical Periodontal Therapy. Med. J. Armed Forces India.

[4] Vijayalashmi R., Ravindranath S.M., Jayakumar N.D., Padmalatha, Vargheese S.H., Kumaraswamy K.L. (2013). Kinetics of drug release from a biodegradable local drug delivery system and its effect on Porphyromonas gingivalis isolates: An in vitro study. J. Indian Soc. Periodontol..

[5] He Z., Liu S., Li Z., Xu J., Liu Y., Luo E. (2022). Coaxial TP/APR electrospun nanofibers for programmed controlling inflammation and promoting bone regeneration in periodontitis-related alveolar bone defect models. Mater. Today Bio..

[6] Deasy P.B., Collins A.E., MacCarthy D.J., Russell R.J. (1989). Use of strips containing tetracycline hydrochloride or metronidazole for the treatment of advanced periodontal disease. J. Pharm. Pharmacol..

[7] Steinberg D., Friedman M., Soskolne A., Sela M.N. (1990). A new degradable controlled release device for treatment of periodontal disease: In vitro release study. J. Periodontol..

[8] Wu L., Chen W., Li F., Morrow B.R., Garcia-Godoy F., Hong L. (2018). Sustained Release of Minocycline from Minocycline-Calcium-Dextran Sulfate Complex Microparticles for Periodontitis Treatment. J. Pharm. Sci..

[9] Pichayakorn W., Boonme P. (2013). Evaluation of cross-linked chitosan microparticles containing metronidazole for periodontitis treatment. Mater. Sci. Eng. C Mater. Biol. Appl..

[10] Xu S., Zhou Q., Jiang Z., Wang Y., Yang K., Qiu X., Ji Q. (2020). The effect of doxycycline-containing chitosan/carboxymethyl chitosan nanoparticles on NLRP3 inflammasome in periodontal disease. Carbohydr. Polym..

[11] Mou J., Liu Z., Liu J., Lu J., Zhu W., Pei D. (2019). Hydrogel containing minocycline and zinc oxide-loaded serum albumin nanopartical for periodontitis application: Preparation, characterization and evaluation. Drug Deliv..

[12] Khorshidi S., Solouk A., Mirzadeh H., Mazinani S., Lagaron J.M., Sharifi S., Ramakrishna S. (2016). A review of key challenges of electrospun scaffolds for tissue-engineering applications. J. Tissue Eng. Regen. Med..

[13] Liu Y., Liu L., Wang Z., Zheng G., Chen Q., Luo E. (2018). Application of Electrospinning Strategy on Cartilage Tissue Engineering. Curr. Stem Cell Res. Ther..

[14] Kouidhi B., Al Qurashi Y.M., Chaieb K. (2015). Drug resistance of bacterial dental biofilm and the potential use of natural compounds as alternative for prevention and treatment. Microb. Pathog..

[15] Lagha A.B., Grenier D. (2019). Tea polyphenols protect gingival keratinocytes against TNF-α-induced tight junction barrier dysfunction and attenuate the inflammatory response of monocytes/macrophages. Cytokine.

[16] Joshi D., Garg T., Goyal A.K., Rath G. (2016). Advanced drug delivery approaches against periodontitis. Drug Deliv..

[17] Esposito E., Cortesi R., Cervellati F., Menegatti E., Nastruzzi C. (1997). Biodegradable microparticles for sustained delivery of tetracycline to the periodontal pocket: Formulatory and drug release studies. J. Microencapsul..

[18] Moreira A.L., Novaes A.B., Grisi M.F., Taba M., Souza S.L., Palioto D.B., de Oliveira P.G., Casati M.Z., Casarin R.C., Messora M.R. (2015). Antimicrobial photodynamic therapy as an adjunct to non-surgical treatment of aggressive periodontitis: A split-mouth randomized controlled trial. J. Periodontol..

[19] Zazo H., Colino C.I., Lanao J.M. (2016). Current applications of nanoparticles in infectious diseases. J. Control. Release.

[20] Shahzad M., Millhouse E., Culshaw S., Edwards C.A., Ramage G., Combet E. (2015). Selected dietary (poly)phenols inhibit periodontal pathogen growth and biofilm formation. Food Funct..

[21] Işılay Özdoğan A., Akca G., Şenel S. (2018). Development and in vitro evaluation of chitosan based system for local delivery of atorvastatin for treatment of periodontitis. Eur. J. Pharm. Sci..

[22] Ahmad N., Ahmad F.J., Bedi S., Sharma S., Umar S., Ansari M.A. (2019). A novel Nanoformulation Development of Eugenol and their treatment in inflammation and periodontitis. Saudi Pharm. J..

[23] Wang B., Shao J., Jansen J.A., Walboomers X.F., Yang F. (2019). A Novel Thermoresponsive Gel as a Potential Delivery System for Lipoxin. J. Dent. Res..

[24] Li X., Luo W., Ng T.W., Leung P.C., Zhang C., Leung K.C., Jin L. (2017). Nanoparticle-encapsulated baicalein markedly modulates pro-inflammatory response in gingival epithelial cells. Nanoscale.

[25] Xu X., Gu Z., Chen X., Shi C., Liu C., Liu M., Wang L., Sun M., Zhang K., Liu Q. (2019). An injectable and thermosensitive hydrogel: Promoting periodontal regeneration by controlled-release of aspirin and erythropoietin. Acta Biomater..

[26] Gunjiganur Vemanaradhya G., Emani S., Mehta D.S., Bhandari S. (2017). Effect of 1.2% of simvastatin gel as a local drug delivery system on Gingival Crevicular Fluid interleukin-6 & interleukin-8 levels in non surgical treatment of chronic periodontitis patients. Arch. Oral Biol..

[27] Soni A., Raj S., Kashyap L., Upadhyay A., Agrahari V.C., Sharma A. (2022). Comparative effect of 1.2% atorvastatin gel and 1.2% rosuvastatin as a local drug delivery in treatment of intra-bony defects in chronic periodontitis. Indian J. Dent. Res..

[28] Manjunatha V.A., Vemanaradhya G.G., Gowda T.M. (2022). Clinical and antioxidant efficacy of 4% mangosteen gel as a local drug delivery in the treatment of chronic periodontitis: A placebo-controlled, split-mouth trial. Dent. Med. Probl..

[29] Nyman S., Lindhe J., Karring T., Rylander H. (1982). New attachment following surgical treatment of human periodontal disease. J. Clin. Periodontol..

[30] Sheikh Z., Qureshi J., Alshahrani A.M., Nassar H., Ikeda Y., Glogauer M., Ganss B. (2017). Collagen based barrier membranes for periodontal guided bone regeneration applications. Odontology.

[31] Wei Y., Deng Y., Ma S., Ran M., Jia Y., Meng J., Han F., Gou J., Yin T., He H. (2021). Local drug delivery systems as therapeutic strategies against periodontitis: A systematic review. J. Control. Release.

[32] Rajeshwari H.R., Dhamecha D., Jagwani S., Rao M., Jadhav K., Shaikh S., Puzhankara L., Jalalpure S. (2019). Local drug delivery systems in the management of periodontitis: A scientific review. J. Control. Release.

[33] Qasim S.S.B., Nogueria L.P., Fawzy A.S., Daood U. (2020). The Effect of Cross-linking Efficiency of Drug-Loaded Novel Freeze Gelated Chitosan Templates for Periodontal Tissue Regeneration. AAPS PharmSciTech.

[34] Liao Y., Li H., Shu R., Chen H., Zhao L., Song Z., Zhou W. (2020). Mesoporous Hydroxyapatite/Chitosan Loaded with Recombinant-Human Amelogenin Could Enhance Antibacterial Effect and Promote Periodontal Regeneration. Front. Cell Infect. Microbiol..

[35] Amato M., Santonocito S., Viglianisi G., Tatullo M., Isola G. (2022). Impact of Oral Mesenchymal Stem Cells Applications as a Promising Therapeutic Target in the Therapy of Periodontal Disease. Int. J. Mol. Sci..

[36] Baba S., Yamada Y., Komuro A., Yotsui Y., Umeda M., Shimuzutani K., Nakamura S. (2016). Phase I/II Trial of Autologous Bone Marrow Stem Cell Transplantation with a Three-Dimensional Woven-Fabric Scaffold for Periodontitis. Stem Cells Int..

[37] Takewaki M., Kajiya M., Takeda K., Sasaki S., Motoike S., Komatsu N., Matsuda S., Ouhara K., Mizuno N., Fujita T. (2017). MSC/ECM Cellular Complexes Induce Periodontal Tissue Regeneration. J. Dent. Res..

[38] Singh Malik D., Mital N., Kaur G. (2016). Topical drug delivery systems: A patent review. Expert Opin. Ther. Pat..

[39] Higashi K., Matsushita M., Morisaki K., Hayashi S., Mayumi T. (1991). Local drug delivery systems for the treatment of periodontal disease. J. Pharmacobiodyn..

[40] Finkelstein A., McClean D., Kar S., Takizawa K., Varghese K., Baek N., Park K., Fishbein M.C., Makkar R., Litvack F. (2003). Local drug delivery via a coronary stent with programmable release pharmacokinetics. Circulation.

[41] Böhme D., Beck-Sickinger A.G. (2015). Drug delivery and release systems for targeted tumor therapy. J. Pept. Sci..

[42] Zhu Y.S., Tang K., Lv J. (2021). Peptide-drug conjugate-based novel molecular drug delivery system in cancer. Trends Pharmacol. Sci..

[43] Qamar Z., Qizilbash F.F., Iqubal M.K., Ali A., Narang J.K., Ali J., Baboota S. (2019). Nano-Based Drug Delivery System: Recent Strategies for the Treatment of Ocular Disease and Future Perspective. Recent Pat. Drug Deliv. Formul..

[44] Sohail M., Guo W., Li Z., Xu H., Zhao F., Chen D., Fu F. (2021). Nanocarrier-based Drug Delivery System for Cancer Therapeutics: A Review of the Last Decade. Curr. Med. Chem..

[45] Adepu S., Ramakrishna S. (2021). Controlled Drug Delivery Systems: Current Status and Future Directions. Molecules.

[46] Kornman K.S. (1993). Controlled-release local delivery antimicrobials in periodontics: Prospects for the future. J. Periodontol..

[47] Goodson J.M., Haffajee A., Socransky S.S. (1979). Periodontal therapy by local delivery of tetracycline. J. Clin. Periodontol..

[48] Allen T.M., Cullis P.R. (2004). Drug delivery systems: Entering the mainstream. Science.

[49] Jain K.K. (2014). Current status and future prospects of drug delivery systems. Methods in Molecular Biology.

[50] Fenton O.S., Olafson K.N., Pillai P.S., Mitchell M.J., Langer R. (2018). Advances in Biomaterials for Drug Delivery. Adv. Mater..

[51] Holborow D., Niederman R., Tonetti M., Cugini M., Goodson J. (1990). Synergistic effects between chlorhexidine mouthwash and tetracycline fibers. J. Dent. Res..

[52] Goodson J.M. (2003). Gingival crevice fluid flow. Periodontol. 2000.

[53] Addy M., Martin M.V. (2003). Systemic antimicrobials in the treatment of chronic periodontal diseases: A dilemma. Oral Dis..

[54] el Kenawy R., Bowlin G.L., Mansfield K., Layman J., Simpson D.G., Sanders E.H., Wnek G.E. (2002). Release of tetracycline hydrochloride from electrospun poly(ethylene-co-vinylacetate), poly(lactic acid), and a blend. J. Control. Release.

[55] Chou S.F., Carson D., Woodrow K.A. (2015). Current strategies for sustaining drug release from electrospun nanofibers. J. Control. Release.

[56] Tonetti M., Cugini M.A., Goodson J.M. (1990). Zero-order delivery with periodontal placement of tetracycline-loaded ethylene vinyl acetate fibers. J. Periodontal. Res..

[57] Zhang Y., Sun T., Jiang C. (2018). Biomacromolecules as carriers in drug delivery and tissue engineering. Acta Pharm. Sin. B.

[58] Kapoor D.N., Bhatia A., Kaur R., Sharma R., Kaur G., Dhawan S. (2015). PLGA: A unique polymer for drug delivery. Ther. Deliv..

[59] Chhina S., Rathore A.S., Juneja S. (2015). Alpha-2-Macroglobulin Levels in Gingival Crevicular Fluid Pre- and Post-scaling and Root Planing with Adjunctive Tetracycline Fibers in Chronic Periodontitis: A Randomized Controlled Trial. J. Contemp. Dent. Pract..

[60] Reise M., Wyrwa R., Müller U., Zylinski M., Völpel A., Schnabelrauch M., Berg A., Jandt K.D., Watts D.C., Sigusch B.W. (2012). Release of metronidazole from electrospun poly(L-lactide-co-D/L-lactide) fibers for local periodontitis treatment. Dent. Mater..

[61] Zhang Z., Zheng Y., Bian X. (2016). Clinical effect of azithromycin as an adjunct to non-surgical treatment of chronic periodontitis: A meta-analysis of randomized controlled clinical trials. J. Periodontal. Res..

[62] Maruyama T., Tomofuji T., Endo Y., Irie K., Azuma T., Ekuni D., Tamaki N., Yamamoto T., Morita M. (2011). Supplementation of green tea catechins in dentifrices suppresses gingival oxidative stress and periodontal inflammation. Arch. Oral. Biol..

[63] Chava V.K., Vedula B.D. (2013). Thermo-reversible green tea catechin gel for local application in chronic periodontitis: A 4-week clinical trial. J. Periodontol..

[64] Assuma R., Oates T., Cochran D., Amar S., Graves D.T. (1998). IL-1 and TNF antagonists inhibit the inflammatory response and bone loss in experimental periodontitis. J. Immunol..

[65] Lee H.A., Song Y.R., Park M.H., Chung H.Y., Na H.S., Chung J. (2020). Catechin ameliorates Porphyromonas gingivalis-induced inflammation via the regulation of TLR2/4 and inflammasome signaling. J. Periodontol..

[66] Wu X., Qiu W., Hu Z., Lian J., Liu Y., Zhu X., Tu M., Fang F., Yu Y., Valverde P. (2019). An Adiponectin Receptor Agonist Reduces Type 2 Diabetic Periodontitis. J. Dent. Res..

[67] Larsen T. (1990). In vitro release of doxycycline from bioabsorbable materials and acrylic strips. J. Periodontol..

[68] Pillai O., Panchagnula R. (2001). Polymers in drug delivery. Curr. Opin. Chem. Biol..

[69] Friesen L.R., Williams K.B., Krause L.S., Killoy W.J. (2002). Controlled local delivery of tetracycline with polymer strips in the treatment of periodontitis. J. Periodontol..

[70] Dixit R.P., Puthli S.P. (2009). Oral strip technology: Overview and future potential. J. Control. Release.

[71] Vyas S.P., Sihorkar V., Mishra V. (2000). Controlled and targeted drug delivery strategies towards intraperiodontal pocket diseases. J. Clin. Pharm. Ther..

[72] Maze G.I., Reinhardt R.A., Agarwal R.K., Dyer J.K., Robinson D.H., DuBois L.M., Tussing G.J., Maze C.R. (1995). Response to intracrevicular controlled delivery of 25% tetracycline from poly(lactide/glycolide) film strips in SPT patients. J. Clin. Periodontol..

[73] Moran J., Addy M., Wade W., Newcombe R. (1990). The use of antimicrobial acrylic strips in the nonsurgical management of chronic periodontitis. Clin. Mater..

[74] Paolantonio M., D’Angelo M., Grassi R.F., Perinetti G., Piccolomini R., Pizzo G., Annunziata M., D’Archivio D., D’Ercole S., Nardi G. (2008). Clinical and microbiologic effects of subgingival controlled-release delivery of chlorhexidine chip in the treatment of periodontitis: A multicenter study. J. Periodontol..

[75] Kudva P., Tabasum S.T., Shekhawat N.K. (2011). Effect of green tea catechin, a local drug delivery system as an adjunct to scaling and root planing in chronic periodontitis patients: A clinicomicrobiological study. J. Indian Soc. Periodontol..

[76] Kashi T.S., Eskandarion S., Esfandyari-Manesh M., Marashi S.M., Samadi N., Fatemi S.M., Atyabi F., Eshraghi S., Dinarvand R. (2012). Improved drug loading and antibacterial activity of minocycline-loaded PLGA nanoparticles prepared by solid/oil/water ion pairing method. Int. J. Nanomed..

[77] Jain R.A. (2000). The manufacturing techniques of various drug loaded biodegradable poly(lactide-co-glycolide) (PLGA) devices. Biomaterials.

[78] Sitarek K., Stetkiewicz J., Wąsowicz W. (2012). Evaluation of reproductive disorders in female rats exposed to N-methyl-2-pyrrolidone. Birth Defects Res. B Dev. Reprod. Toxicol..

[79] Ruan H., Yu Y., Liu Y., Ding X., Guo X., Jiang Q. (2016). Preparation and characteristics of thermoresponsive gel of minocycline hydrochloride and evaluation of its effect on experimental periodontitis models. Drug Deliv..

[80] Oliveira M.S., Goulart G.C.A., Ferreira L.A.M., Carneiro G. (2017). Hydrophobic ion pairing as a strategy to improve drug encapsulation into lipid nanocarriers for the cancer treatment. Expert Opin. Drug Deliv..

[81] Lu H.D., Rummaneethorn P., Ristroph K.D., Prud’homme R.K. (2018). Hydrophobic Ion Pairing of Peptide Antibiotics for Processing into Controlled Release Nanocarrier Formulations. Mol. Pharm..

[82] Holmkvist A.D., Friberg A., Nilsson U.J., Schouenborg J. (2016). Hydrophobic ion pairing of a minocycline/Ca(2+)/AOT complex for preparation of drug-loaded PLGA nanoparticles with improved sustained release. Int. J. Pharm..

[83] Bhattarai N., Gunn J., Zhang M. (2010). Chitosan-based hydrogels for controlled, localized drug delivery. Adv. Drug Deliv. Rev..

[84] Gad H.A., Kamel A.O., Ezzat O.M., El Dessouky H.F., Sammour O.A. (2017). Doxycycline hydrochloride-metronidazole solid lipid microparticles gels for treatment of periodontitis: Development, in-vitro and in-vivo clinical evaluation. Expert Opin. Drug Deliv..

[85] Mehnert W., Mäder K. (2001). Solid lipid nanoparticles: Production, characterization and applications. Adv. Drug Deliv. Rev..

[86] Jadhav K., Dhamecha D., Bhattacharya D., Patil M. (2016). Green and ecofriendly synthesis of silver nanoparticles: Characterization, biocompatibility studies and gel formulation for treatment of infections in burns. J. Photochem. Photobiol. B.

[87] Park E.J., Yi J., Kim Y., Choi K., Park K. (2010). Silver nanoparticles induce cytotoxicity by a Trojan-horse type mechanism. Toxicol. In Vitro.

[88] Inkielewicz-Stepniak I., Santos-Martinez M.J., Medina C., Radomski M.W. (2014). Pharmacological and toxicological effects of co-exposure of human gingival fibroblasts to silver nanoparticles and sodium fluoride. Int. J. Nanomed..

[89] Yao W., Xu P., Pang Z., Zhao J., Chai Z., Li X., Li H., Jiang M., Cheng H., Zhang B. (2014). Local delivery of minocycline-loaded PEG-PLA nanoparticles for the enhanced treatment of periodontitis in dogs. Int. J. Nanomed..

[90] Kaya M., Baran T., Erdoğan S., Menteş A., Özüsağlam M.A., Çakmak Y.S. (2014). Physicochemical comparison of chitin and chitosan obtained from larvae and adult Colorado potato beetle (Leptinotarsa decemlineata). Mater. Sci. Eng. C Mater. Biol. Appl..

[91] Moura L.A., Ribeiro F.V., Aiello T.B., Duek E.A., Sallum E.A., Nociti Junior F.H., Casati M.Z., Sallum A.W. (2015). Characterization of the release profile of doxycycline by PLGA microspheres adjunct to non-surgical periodontal therapy. J. Biomater. Sci. Polym. Ed..

[92] Sgolastra F., Petrucci A., Severino M., Graziani F., Gatto R., Monaco A. (2013). Adjunctive photodynamic therapy to non-surgical treatment of chronic periodontitis: A systematic review and meta-analysis. J. Clin. Periodontol..

[93] Alwaeli H.A., Al-Khateeb S.N., Al-Sadi A. (2015). Long-term clinical effect of adjunctive antimicrobial photodynamic therapy in periodontal treatment: A randomized clinical trial. Lasers Med. Sci..

[94] Sreedhar A., Sarkar I., Rajan P., Pai J., Malagi S., Kamath V., Barmappa R. (2015). Comparative evaluation of the efficacy of curcumin gel with and without photo activation as an adjunct to scaling and root planing in the treatment of chronic periodontitis: A split mouth clinical and microbiological study. J. Nat. Sci. Biol. Med..

[95] de Freitas L.M., Calixto G.M., Chorilli M., Giusti J.S., Bagnato V.S., Soukos N.S., Amiji M.M., Fontana C.R. (2016). Polymeric Nanoparticle-Based Photodynamic Therapy for Chronic Periodontitis in Vivo. Int. J. Mol. Sci..

[96] Baelo A., Levato R., Julián E., Crespo A., Astola J., Gavaldà J., Engel E., Mateos-Timoneda M.A., Torrents E. (2015). Disassembling bacterial extracellular matrix with DNase-coated nanoparticles to enhance antibiotic delivery in biofilm infections. J. Control. Release.

[97] Forier K., Raemdonck K., De Smedt S.C., Demeester J., Coenye T., Braeckmans K. (2014). Lipid and polymer nanoparticles for drug delivery to bacterial biofilms. J. Control. Release.

[98] Pérez-Pacheco C.G., Fernandes N.A.R., Primo F.L., Tedesco A.C., Bellile E., Retamal-Valdes B., Feres M., Guimarães-Stabili M.R., Rossa C. (2021). Local application of curcumin-loaded nanoparticles as an adjunct to scaling and root planing in periodontitis: Randomized, placebo-controlled, double-blind split-mouth clinical trial. Clin. Oral. Investig..

[99] Izui S., Sekine S., Maeda K., Kuboniwa M., Takada A., Amano A., Nagata H. (2016). Antibacterial Activity of Curcumin Against Periodontopathic Bacteria. J. Periodontol..

[100] Zambrano L.M.G., Brandao D.A., Rocha F.R.G., Marsiglio R.P., Longo I.B., Primo F.L., Tedesco A.C., Guimaraes-Stabili M.R., Rossa Junior C. (2018). Local administration of curcumin-loaded nanoparticles effectively inhibits inflammation and bone resorption associated with experimental periodontal disease. Sci. Rep..

[101] Zhou T., Chen D., Li Q., Sun X., Song Y., Wang C. (2013). Curcumin inhibits inflammatory response and bone loss during experimental periodontitis in rats. Acta Odontol. Scand..

[102] Perez-Pacheco C.G., Fernandes N.A.R., Camilli A.C., Ferrarezi D.P., Silva A.F., Zunareli M.C., Amantino C.F., Primo F.L., Guimarães-Stabilli M.R., Junior C.R. (2022). Local administration of curcumin-loaded nanoparticles enhances periodontal repair in vivo. Naunyn Schmiedebergs Arch. Pharmacol..

[103] Allen T.M., Cullis P.R. (2013). Liposomal drug delivery systems: From concept to clinical applications. Adv. Drug Deliv. Rev..

[104] Thangavelu A., Stelin K.S., Vannala V., Mahabob N., Hayyan F.M.B., Sundaram R. (2021). An Overview of Chitosan and Its Role in Periodontics. J. Pharm. Bioallied. Sci..

[105] Akncbay H., Senel S., Ay Z.Y. (2007). Application of chitosan gel in the treatment of chronic periodontitis. J. Biomed. Mater. Res. B Appl. Biomater..

[106] Ikinci G., Senel S., Akincibay H., Kaş S., Erciş S., Wilson C.G., Hincal A.A. (2002). Effect of chitosan on a periodontal pathogen Porphyromonas gingivalis. Int. J. Pharm..

[107] Dong W.Y., Körber M., López Esguerra V., Bodmeier R. (2006). Stability of poly(D,L-lactide-co-glycolide) and leuprolide acetate in in-situ forming drug delivery systems. J. Control. Release.

[108] Ibrahim H.M., Ahmed T.A., Hussain M.D., Rahman Z., Samy A.M., Kaseem A.A., Nutan M.T. (2014). Development of meloxicam in situ implant formulation by quality by design principle. Drug Dev. Ind. Pharm..

[109] Wang B., Wang J., Shao J., Kouwer P.H.J., Bronkhorst E.M., Jansen J.A., Walboomers X.F., Yang F. (2020). A tunable and injectable local drug delivery system for personalized periodontal application. J. Control. Release.

[110] Lu S., Ren X., Guo T., Cao Z., Sun H., Wang C., Wang F., Shu Z., Hao J., Gui S. (2021). Controlled release of iodine from cross-linked cyclodextrin metal-organic frameworks for prolonged periodontal pocket therapy. Carbohydr. Polym..

[111] Messora M.R., Apolinário Vieira G.H., Vanderlei J., Mariguela V.C., Fernandes P.G., Palioto D.B., Scombatti de Souza S.L., Novaes A.B., Furlaneto F., Taba M. (2017). Rosuvastatin promotes benefits on induced periodontitis in hypertensive rats. J. Periodontal. Res..

[112] Lim S.Y., Dafydd M., Ong J., Ord-McDermott L.A., Board-Davies E., Sands K., Williams D., Sloan A.J., Heard C.M. (2020). Mucoadhesive thin films for the simultaneous delivery of microbicide and anti-inflammatory drugs in the treatment of periodontal diseases. Int. J. Pharm..

[113] Lizambard M., Menu T., Fossart M., Bassand C., Agossa K., Huck O., Neut C., Siepmann F. (2019). In-situ forming implants for the treatment of periodontal diseases: Simultaneous controlled release of an antiseptic and an anti-inflammatory drug. Int. J. Pharm..

[114] de Santana R.B., de Santana C.M. (2015). Human intrabony defect regeneration with rhFGF-2 and hyaluronic acid—A randomized controlled clinical trial. J. Clin. Periodontol..

[115] Qi Y., Yang J., Chi Y., Wen P., Wang Z., Yu S., Xue R., Fan J., Li H., Chen W. (2022). Natural polyphenol self-assembled pH-responsive nanoparticles loaded into reversible hydrogel to inhibit oral bacterial activity. Mol. Biomed..

[116] Liang J., Peng X., Zhou X., Zou J., Cheng L. (2020). Emerging Applications of Drug Delivery Systems in Oral Infectious Diseases Prevention and Treatment. Molecules.

[117] Gottlow J. (1993). Guided Tissue Regeneration Using Bioresorbable and Non-Resorbable Devices: Initial Healing and Long-Term Results. J. Periodontol..

[118] Bunyaratavej P., Wang H.L. (2001). Collagen membranes: A review. J. Periodontol..

[119] Behring J., Junker R., Walboomers X.F., Chessnut B., Jansen J.A. (2008). Toward guided tissue and bone regeneration: Morphology, attachment, proliferation, and migration of cells cultured on collagen barrier membranes. A systematic review. Odontology.

[120] Liu X., Zhang W., Wang Y., Chen Y., Xie J., Su J., Huang C. (2020). One-step treatment of periodontitis based on a core-shell micelle-in-nanofiber membrane with time-programmed drug release. J. Control. Release.

[121] Ho M.H., Claudia J.C., Tai W.C., Huang K.Y., Lai C.H., Chang C.H., Chang Y.C., Wu Y.C., Kuo M.Y., Chang P.C. (2021). The treatment response of barrier membrane with amoxicillin-loaded nanofibers in experimental periodontitis. J. Periodontol..

[122] Carlson-Mann L.D., Ibbott C.G., Grieman R.B. (1996). Ridge augmentation with guided bone regeneration and GTAM case illustrations. Probe.

[123] Ma W., Lyu H., Pandya M., Gopinathan G., Luan X., Diekwisch T.G.H. (2021). Successful Application of a Galanin-Coated Scaffold for Periodontal Regeneration. J. Dent. Res..

[124] Ouchi T., Nakagawa T. (2020). Mesenchymal stem cell-based tissue regeneration therapies for periodontitis. Regen. Ther..

[125] Genco R.J., Sanz M. (2020). Clinical and public health implications of periodontal and systemic diseases: An overview. Periodontol. 2000.

[126] O’Connell P.A., Taba M., Nomizo A., Foss Freitas M.C., Suaid F.A., Uyemura S.A., Trevisan G.L., Novaes A.B., Souza S.L., Palioto D.B. (2008). Effects of periodontal therapy on glycemic control and inflammatory markers. J. Periodontol..

[127] Faria-Almeida R., Navarro A., Bascones A. (2006). Clinical and metabolic changes after conventional treatment of type 2 diabetic patients with chronic periodontitis. J. Periodontol..

[128] Matesanz-Pérez P., García-Gargallo M., Figuero E., Bascones-Martínez A., Sanz M., Herrera D. (2013). A systematic review on the effects of local antimicrobials as adjuncts to subgingival debridement, compared with subgingival debridement alone, in the treatment of chronic periodontitis. J. Clin. Periodontol..

[129] Lecio G., Ribeiro F.V., Pimentel S.P., Reis A.A., da Silva R.V.C., Nociti F., Moura L., Duek E., Casati M., Casarin R.C.V. (2020). Novel 20% doxycycline-loaded PLGA nanospheres as adjunctive therapy in chronic periodontitis in type-2 diabetics: Randomized clinical, immune and microbiological trial. Clin. Oral. Investig..

[130] Pereira A., Brito G.A.C., Lima M.L.S., Silva Júnior A.A.D., Silva E.D.S., de Rezende A.A., Bortolin R.H., Galvan M., Pirih F.Q., Araújo Júnior R.F. (2018). Metformin Hydrochloride-Loaded PLGA Nanoparticle in Periodontal Disease Experimental Model Using Diabetic Rats. Int. J. Mol. Sci..

[131] Giménez-Siurana A., Gómez García F., Pagan Bernabeu A., Lozano-Pérez A.A., Aznar-Cervantes S.D., Cenis J.L., López-Jornet P. (2020). Chemoprevention of Experimental Periodontitis in Diabetic Rats with Silk Fibroin Nanoparticles Loaded with Resveratrol. Antioxidants.

[132] de Souza Malta F., Napimoga M.H., Marins L.M., Miranda T.S., de Oliveira F.B., Posch A.T., Feres M., Duarte P.M. (2020). Lithium chloride assuages bone loss in experimental periodontitis in estrogen-deficient rats. Clin. Oral. Investig..

[133] Cardoso R.S., Messora M.R., Silva P.H.F., Oliveira L.F., Leite-Panissi C., Salvador S., Casarin R., Novaes A.B., Palioto D.B., Furlaneto F.A.C. (2020). Effects of Bifidobacterium animalis subsp. lactis HN019 on ligature-induced periodontitis in rats with experimental rheumatoid arthritis. Benef. Microbes.

[134] Boese S., Gill H.S. (2021). Coated floss for drug delivery into the gum pocket. Int. J. Pharm..

